# Colorectal laterally spreading tumors show characteristic expression of cell polarity factors, including atypical protein kinase C λ/ι, E-cadherin, β-catenin and basement membrane component

**DOI:** 10.3892/ol.2014.2271

**Published:** 2014-06-20

**Authors:** YASUSHI ICHIKAWA, YOJI NAGASHIMA, KAORI MORIOKA, KAZUNORI AKIMOTO, YASUYUKI KOJIMA, TAKASHI ISHIKAWA, AYUMU GOTO, NORITOSHI KOBAYASHI, KAZUTERU WATANABE, MITSUYOSHI OTA, SHOICHI FUJII, MAYUMI KAWAMATA, RYO TAKAGAWA, CHIKARA KUNIZAKI, HIROKAZU TAKAHASHI, ATSUSHI NAKAJIMA, SHIN MAEDA, HIROSHI SHIMADA, YOSHIAKI INAYAMA, SHIGEO OHNO, ITARU ENDO

**Affiliations:** 1 Department of Clinical Oncology, Yokohama City University Graduate School of Medicine, Yokohama, Kanagawa 23600044, Japan; 2 Department of Molecular Pathology, Yokohama City University Graduate School of Medicine, Yokohama, Kanagawa 23600044, Japan; 3 Department of Gastroenterological Surgery, Yokohama City University Graduate School of Medicine, Yokohama, Kanagawa 23600044, Japan; 4 Department of Molecular Biology, Yokohama City University Graduate School of Medicine, Yokohama, Kanagawa 23600044, Japan; 5 Department of Gastroenterology, Yokohama City University Graduate School of Medicine, Yokohama, Kanagawa 23600044, Japan

**Keywords:** laterally spreading tumor, atypical protein kinase C λ/ι

## Abstract

Colorectal flat-type tumors include laterally spreading tumors (LSTs) and flat depressed-type tumors. The former of which shows a predominant lateral spreading growth rather than an invasive growth. The present study examined the morphological characteristics of LSTs, in comparison with polypoid- or flat depressed-type tumors, along with the expression of atypical protein kinase C (aPKC) λ/ι, a pivotal cell polarity regulator, and the hallmarks of cell polarity, as well as with type IV collagen, β-catenin and E-cadherin. In total, 37 flat-type (24 LSTs and 13 flat depressed-type tumors) and 20 polypoid-type colorectal tumors were examined. The LSTs were classified as 15 LST adenoma (LST-A) and nine LST cancer in adenoma (LST-CA). An immunohistochemical examination was performed on aPKC λ/ι, type IV collagen, β-catenin and E-cadherin. The LST-A and -CA showed a superficial replacing growth pattern, with expression of β-catenin and E-cadherin in the basolateral membrane and type IV collagen along the basement membrane. In addition, 86.6% of LST-A and 55.6% of LST-CA showed aPKC λ/ι expression of 1+ (weak to normal intensity staining in the cytoplasm compared with the normal epithelium). Furthermore, ~45% of the polypoid-type adenomas showed 2+ (moderate intensity staining in the cytoplasm and/or nucleus) and 66.7% of the polypoid-type cancer in adenoma were 3+ (strong intensity staining in the cytoplasm and nucleus). A statistically significant positive correlation was observed between the expression of aPKC λ/ι and β-catenin (r=0.842; P<0.001), or type IV collagen (r=0.823; P<0.001). The LSTs showed a unique growth pattern, different from the expanding growth pattern presented by a polypoid tumor and invasive cancer. The growth characteristics of LST appear to be caused by adequate coexpression of β-catenin, type IV collagen and aPKC λ/ι.

## Introduction

The well-organized architectures of the normal colonic epithelia are inevitably associated with the apical and basolateral polarity (1). The basolateral polarity of the normal epithelium is maintained by conservation of the basement membrane (BM) between the cells and the extracellular matrix (ECM), as well as the expression of adhesion molecules on the plasma membranes, including E-cadherin and β-catenin, between the epithelial cells. In certain situations, the cell polarity is disturbed and the remodeling of the epithelial cell organization, including wound healing and cancer progression, is required. In these processes, the epithelial cells obtain increased motility showing front-rear polarity similar to that of mesenchymal cells, instead of apical and basolateral polarity.

Epithelial cell polarity is regulated by highly conserved polarity proteins and the atypical protein kinase C (aPKC) is a protein family that is one of the most important signaling components controlling cell polarity. In particular, aPKC λ/ι is a pivotal regulator of cell polarity and has been reported to be associated with the pathogenesis and progression of neoplasms (1). Changes in aPKC λ/ι expression have been reported in several types of tumors and the overexpression of aPKC λ/ι is associated with the progression and prognosis of various carcinomas (2–5).

Laterally spreading tumors (LSTs) are flat-type colorectal tumors that are gross morphological concepts in contrast to polypoid-type tumors. Kudo (6) defined LSTs as colorectal tumors growing superficially along the mucosal surface with a short vertical length despite horizontal diameters of >10 mm. Their superficial replacing growth is confirmed by light microscopy and, according to technical improvements in endoscopy, an increased number of LSTs have been diagnosed and resected. The majority of LSTs are histologically adenoma, however, several cases have been identified as cancerous. Notably, the majority of cancerous LSTs also show superficial replacing growth and less invasive behavior. A flat depressed-type tumor is an additional type of non-polypoid colorectal neoplasm (7), which shows expanding growth and massive submucosal invasion in early-stage cancer in comparison with LST (8,9). Protruded-type tumors also show expanding growth and, therefore, the cell polarity of LSTs approaches that of the normal mucosal epithelia. However, flat depressed- or protruded-type tumors also show invasive growth.

These observations suggested that there may be a difference between LST and flat depressed- and polypoid-type lesions in the cell polarity status. These results prompted an investigation of the expression and localization of cell polarity-related proteins in LST, as well as in flat depressed-and polypoid-type lesions, using four immunomarkers against aPKC λ/ι, β-catenin, E-cadherin and type IV collagen.

## Materials and methods

### Samples

In total, 37 flat-type and 20 polypoid-type colorectal tumors were selected. All of the lesions were endoscopically or surgically resected at the Yokohama City University Hospital (Yokohama, Japan), between 1998 and 2011. The resected tissue was immediately fixed in 20% formalin and embedded in paraffin. Next, 4-μm thick paraffin sections were stained with hematoxylin and eosin and subjected to pathological diagnosis. The flat-type tumors included 15 adenomas (LST, adenoma; LST-A), nine non-invasive adenocarcinoma in adenomas (LST, cancer in adenomas; LST-CAs) and 13 flat depressed-type tumors. The polypoid-type tumors included 11 adenomas (polypoid-type adenomas; P-As) and nine non-invasive adenocarcinoma in adenomas (polypoid-type cancer in adenomas; P-CAs) (Table IA and B). The flat depressed-type cancers (FD-CAs) were all invasive cancers. The study was approved by the institutional ethical committee, Yokohama City University Ethical Review Board (Yokohama, Japan) and written informed consent was obtained from all the enrolled patients for the use of the samples.

### Immunohistochemistry

The expression and localization of aPKC λ/ι, β-catenin, E-cadherin and type IV collagen were immunohistochemically examined as previously described (2,3). Briefly, 4 μm-thick paraffin sections were deparaffinized and rehydrated. Next, the antigen retrieval was performed by autoclaving (for aPKC λ/ι), microwaving three times for 3 min each time (for β-catenin and E-cadherin) or digestion with proteinase K (0.4 mg/ml; DakoCytomation, Glostrup, Denmark) at room temperature for 6 to 15 min (for type IV collagen). The endogenous peroxidase activity was quenched by immersing the sections in 0.3% hydrogen peroxide/phosphate-buffered saline for 30 min at room temperature, and the sections were incubated with 10% goat serum (Pierce Biotechnology, Inc., Rockford, IL, USA) at room temperature for 20 min to block non-specific protein binding. The primary antibodies were applied to the sections and incubated for 1 h at room temperature for type IV collagen, and overnight at 4°C for aPKC λ/ι, β-catenin and E-cadherin staining. The monoclonal mouse anti-human antibodies against aPKC λ/ι (clone 23/PKCi; cat. no. 610176; BD Transduction Laboratories, Lexington, KY, USA), β-catenin (clone 14; 1:100; BD Transduction Laboratories), E-cadherin (BV-6; 1:100; Chemicon, Temecula, CA, USA) and type IV collagen (CIV 22; 1:50; DakoCytomation) were used as primary antibodies. The labeled antigens were visualized by the HistoFine kit (Nichirei, Tokyo, Japan) followed by 3,3′-diaminobenzidine reaction. The sections were then counterstained with hematoxylin and microscopically observed (Olympus BX41; Olympus Corporation, Tokyo, Japan).

### Evaluation of aPKC λ/ι expression

The intensities of the immunopositive signals for aPKC λ/ι in the neoplasms were semi-quantitatively scored by one pathologist not blinded to the study according to the following previously employed criteria (2): 1+, weak to normal intensity staining in the cytoplasm in comparison to the normal epithelium; 2+, moderate intensity staining in the cytoplasm and/or nucleus; and 3+, strong intensity staining in the cytoplasm and nucleus.

### Evaluation of β-catenin and E-cadherin expression

The expression of β-catenin and E-cadherin in the cancer cells was compared with that of the normal epithelial cells as a standard, as normal epithelial cells exhibit strong expression of these proteins at the intercellular boundaries. The expression of β-catenin in the colorectal tumors was classified into the following three subclasses: i) Preserved type, staining localized on the cell surface membrane; ii) cytoplasmic type, diffuse cytoplasmic staining; and iii) nucleic type, nuclear staining (10). E-cadherin expression was also classified into three subclasses according to Oka *et al* (11), as follows: i) Preserved type, ≥90% of neoplastic cells positive for E-cadherin; ii) heterogeneous type,>10 and <90%; and iii) lost type, ≤10%.

### Evaluation of type IV collagen expression

Type IV collagen expression in colorectal tumors was classified into the following three subclasses: i) Continuous type, continuous linear staining in the BM of glands; ii) discontinuous type, discontinuous staining in the BM of glands; and iii) lost type, no staining in the BM of glands (12).

### Statistical analysis

Statistical analyses were performed using the SPSS program version 17 for Windows (SPSS, Inc., Chicago, IL, USA). The differences in the expression patterns of the antigens between all of the tumor groups were compared using the χ^2^ test, and χ^2^ tests with Fisher’s exact correction were applied when the incidence was <5. To analyze the correlation between the expression intensity of aPKC λ/ι staining and other staining, Pearson’s correlation coefficient (r) was used.

## Results

### Histopathological characteristics of colon tumors

**Figure 1:**
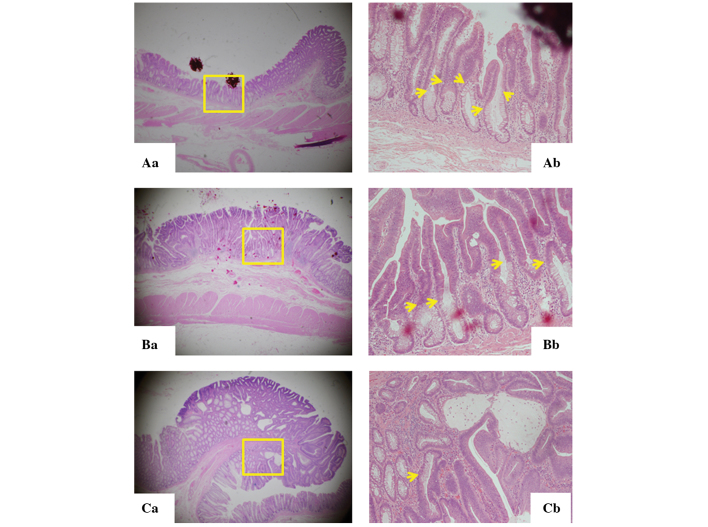
Hematoxylin and eosin staining of LSTs and P-As. (A) LST-As showed multiple borders of tumor and normal epithelium. A cancer front, at multiple sites in the tumor (as indicated by the arrows in Ab) of the LST-A appears to exhibit two layers composed of a tumorous layer on the apical side and a normal layer on the under side. (B) The same structure was detected in cancer *in situ* of LST (as indicated by the arrows in Bb) showing two layers of normal tissue on the under side and apical side of the tumor. (C) The cancer front of the P-As and depressed-type tumors were detected only on the border of the tumors (as indicated by the arrows in Cb), but no two layer structures were identified. Magnifications of (Aa, Ba and Ca) ×4 and (Ab, Bb and Cb) ×100. The yellow boxes represent the magnified areas shown in Ab, Bb and Cb. LST, laterally spreading tumor; P-A, polypoid-type adenoma.

LST-As exhibited evident boundaries with the normal epithelium, known as the lesional front, at multiple sites in the lesion (Fig. 1Aa and Ab). The LST-A occupies the surface mucosa with bottom-situated normal mucosa, presenting a two-layered elevated lesion. The same architecture was observed in LST-CAs showing the top layer of cancer in adenoma and the bottom layer of normal tissue (Fig. 1Ba and Bb). However, the tumor front of polypoid-type tumors and FD-CAs was detected only in the border of tumors, and they did not show the two-layered structures (Fig. 1Ca and Cb).

### Expression of aPKC λ/ι

**Figure 2:**
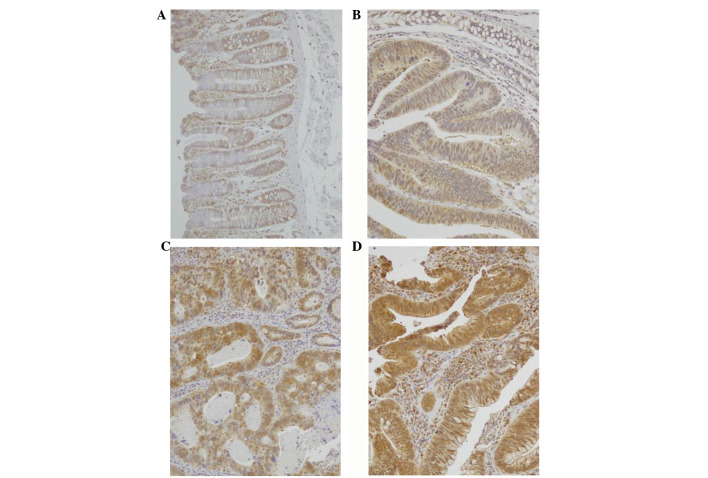
Expression of atypical protein kinase C λ/ι. (A) The cytoplasm and nucleus were marginally stained in the normal mucosa. (B) Weak to normal intensity staining in the cytoplasm of the normal epithelium (1+). (C) Moderate intensity staining in the cytoplasm and/or nucleus of polypoid cancer in adenoma (2+). (D) Strong intensity staining in the cytoplasm and nucleus of depressed-type cancer (3+) (magnification, ×400).

**Figure 3:**
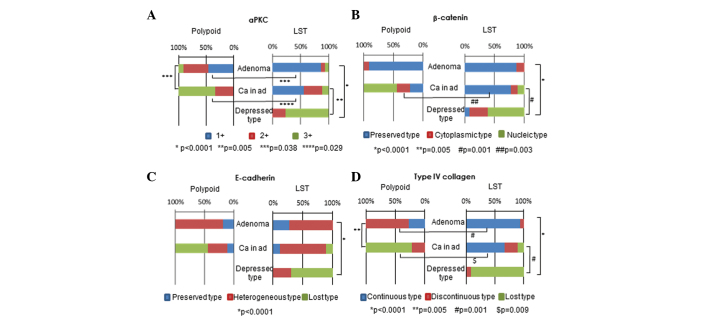
(A) Expression of aPKC was compared among five types of tumors. The χ^2^ test or χ^2^ test with Fisher’s exact correction was applied to the ratio of 1+ vs. 2+ or 3+ for each tumor type. LST-As showed an extremely high ratio of aPKC 1+ compared with P-As and FD-CAs. This distinction was also observed in LST-CAs. P-CAs and FD-CAs did not show aPKC 1+; however, 55.6% of LST-CAs showed aPKC 1+. (B) Expression of β-catenin was compared among five types of tumor. The ratio of the preserved type was ~90% in the LST-As and P-As. The ratio was lower in P-CAs and the FD-CAs, at 22.2 and 7.7%, respectively, and LST-CAs showed a statistically higher ratio (77.8%) than the other two groups. (C) The expression of E-cadherin was compared among five types of tumors. E-cadherin was not preserved in all five groups and the ratio of the lost type was high in the P-CAs and FD-CAs. (D) The expression of type IV collagen was compared among five types of tumor. The ratio of the continuous type was significantly higher in LST-As and -CAs than in the P-As, P-CAs and FD-CAs. aPKC, atypical protein kinase C; LST-A, laterally spreading tumor, adenoma; LST-CA, LST, cancer in adenoma; FD-CA, flat depressed-type cancer; P-A, polypoid-type adenoma; P-CA, polypoid-type cancer in adenoma.

Representative images of the various intensities of immunostaining for aPKC λ/ι, 1+ to 3+, are shown in Fig. 2B–D, respectively. As shown in Table IIA, the intensities of aPKC λ/ι immunostaining were 1+ in 86.6% of LST-As and 2+ in 45.5% of P-As. Additionally, ~70% of P-CAs and FD-CAs were 3+ . On the other hand, ~55.6% of LST-CAs were 1+, and LST-As and LST-CAs showed significantly lower expression of aPKC λ/ι than P-As or P-CAs (P=0.038 and 0.029, respectively; Fig. 3A, Table IIA).

### Expression of β-catenin, E-cadherin and type IV collagen

**Figure 4:**
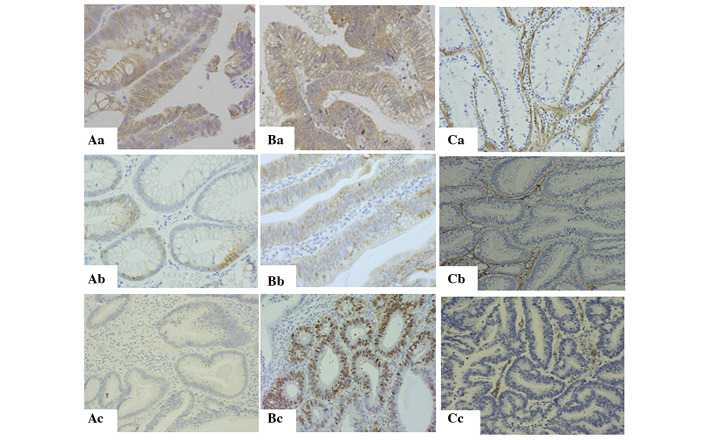
Expression of E-cadherin, β-catenin and type IV collagen by immunohistochemistry. Expression of E-cadherin was (Aa) detected on the surface of the cell membrane in >90% of the epithelial cells (preserved type), (Ab) partially detected in the cell membrane or cytoplasm (heterogeneous type) and (Ac) not detected in the basement membrane of the glands (lost type). Expression of β-catenin was detected in the (Ba) surface of the cell membrane (preserved type), (Bb) cytoplasm (cytoplasmic type) and (Ac) nucleus (nucleic type). Expression of type IV collagen showed (Ca) continuous expression in laterally spreading tumor adenoma (continuous type), which indicated that the basement membrane was completely conserved, (Cb) partial lack of expression in polypoid-type adenoma (discontinuous type) around the gland and (Cc) no expression in invasive cancer of flat depressed type (lost type).

Fig. 3B–D and Table IIB-D summarize the expression of β-catenin, E-cadherin and type IV collagen, respectively, in LSTs and polypoid-type tumors of each histological type. The results showed that 86.6% of the LST-As and 99% of the P-As (Fig. 3B, Table IIB) showed the preserved type of expression of β-catenin (Fig. 4Aa). Furthermore, 77.8% of LST-CAs also showed the preserved type of expression. On the other hand, 55.6% of P-CAs and 61.5% of FD-CAs showed the nucleic type (Fig. 4Ac). The expression of E-cadherin in the preserved type (Fig. 4Ba) was only observed in 26.7% of LST-As, 18.2% of P-As and 11.1% of LST-CAs and P-As (Fig. 3C, Table IIC). The expression of type IV collagen was observed in 93.3% of LST-As (Fig. 3D, Table IID); however, only 27.3% of P-As were of the continuous type (Fig. 4Ca). In addition, 92.4% of FD-CAs and 77.8% of P-CAs were of the lost type (Fig. 4Cc). Notably, 66.7% of LST-CAs showed the continuous type (Fig. 4Cb).

**Figure 5:**
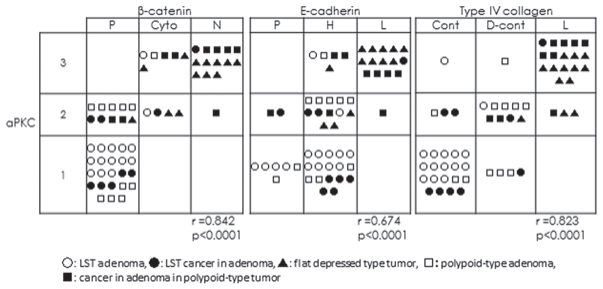
Correlation between aPKC λ/ι and β-catenin, E-cadherin and type IV collagen. The expression of aPKC λ/ι showed a strong positive correlation with that of β-catenin (r=0.842) and type IV collagen (r=0.823), however, a weaker positive correlation was identified between aPKC λ/ι and E-cadherin expression (r=0.674) (P<0.0001 for all). aPKC, atypical protein kinase C; r, Pearson’s correlation coefficient; LST, laterally spreading tumor; P, preserved type; Cyto, cytoplasmic type; N, nucleic type; H, heterogeneous type; L, lost type; Cont, continuous type; D-cont, discontinuous type.

Pearson’s correlation coefficient using the expression results of all the samples showed a significant positive correlation between the expression of aPKC λ/ι and β-catenin (r=0.842; P<0.001) and type IV collagen (r=0.823; P<0.001) (Fig. 5). A significant correlation was identified between PKC and E-cadherin, but a marginally weaker positive correlation/r value was identified (r=0.674; P<0.001; Fig. 5).

## Discussion

The present study showed the unique morphological and functional characteristics of cell polarity proteins in LSTs, including adenoma and cancer in adenoma.

LSTs of the colon and rectum are morphologically defined as lesions of >10 mm in diameter with a low vertical axis that extend laterally along the luminal wall. There are two macroscopic subtypes of LST: G type, with a granule aggregating surface (13); and NG type, with a flat, smooth and non-granule aggregating surface. The majority of LSTs remain as adenomas or early invasive cancers, thus LSTs are considered to have potentially carcinogenic, but less invasive characteristics (8,14). These characteristics can be identified in the LST’s growth morphology as neoplastic cells that tend to spread along the surface of the lumen. The microscopic image in Fig. 1Ab and Bb, showing the of the LST’s two layers, revealed a unique growth morphology, which is maintained in cancerous LST lesions (4). By contrast, polypoid-type tumors, another type of colorectal tumor, show expanding growth. The two types of tumor show not only morphological differences, but also some genetic or epigenetic differences (15). An additional flat type of colorectal tumor besides LSTs is the flat depressed-type tumor (types IIc, IIc+IIa and or IIa+IIc), shows more invasive characteristics than LSTs. Ohno *et al* showed that flat depressed-type tumors also show a low vertical axis that extends laterally along the luminal wall similar to LSTs (8); however, they show an expanding growth and do not exhibit two layers. Furthermore, the authors also showed that ~70% of cancerous lesions in LSTs are cancer *in situ* and *~*64% of cancerous lesions of flat depressed-type tumors show massive submucosal invasion (8). Certain types of LSTs become flat depressed-type tumors in the course of cancer progression and the surface spreading growth pattern reveals that LSTs show the expansive and invasive growth pattern of flat depressed-type tumors during that progression.

Notably, the current study results showed that LST-CAs maintain β-catenin and E-cadherin expression in the cell membrane and that the BM was maintained around the tumor. However, P-CAs and FD-CAs lost the expression of the proteins and the BM. The BM structure was already lost in P-As.

The epithelial structure of colorectal mucosa has apical and basolateral polarity (16), and the basal pole corresponds to the contact between the cell membrane and extracellular BM molecules (17). The BM is an important structure that determines whether epithelial cells are aligned on the ECM or migrate into it. No BM abnormalities have been noted in hyperplastic polyps; however, discrete disruption in the BM may be found in adenoma, depending on the degree of epithelial atypia (18). Colorectal adenocarcinoma shows various BM patterns and the majority of cancer cell nests do not exhibit any BM. However, the current study found that non-invasive cancer cells in LST structures have almost normal BM structures.

E-cadherin and β-catenin are necessary for the cell-cell adhesion of normal colonic epithelial cells (19) and are important factors that determine the lateral pole of colonic epithelial cells. The loss of the staining pattern and lower level or absence of E-cadherin expression in colorectal tumors is associated with increasing histological grading and worse prognosis (20). β-catenin forms a complex with glycogen synthase kinase 3β, adenomatous polyposis coli (APC) and axis inhibition protein, which binds with a T-cell factor in the nucleus to promote gene transcription and contribute to colorectal carcinogenesis (21). The distribution of nuclear β-catenin expression is utilized as a prognostic marker in colorectal cancer (22). Hashimoto *et al* (23) showed that β-catenin in LSTs is expressed more intensely in flat structure segments or invasive lesions than in granulation structures or intramucosal lesions. Wang *et al* (24) also reported that β-catenin is expressed more prominently in LSTs than in protruded-type adenoma. The authors evaluated the β-catenin expression by counting stained cells and did not report the distribution of β-catenin in the nucleus, cytoplasm or cell membrane. The results of the current study showed that E-cadherin and the expression of β-catenin were maintained in the cell membrane in LST-As and LST-CAs, and that the expression was lost or exhibited abnormal distribution in polypoid-type tumors.

Cell polarity is regulated by complex systems and Par-6, Par-3 and aPKC are major regulators of basolateral polarity (25). These regulators are involved in tight junction-associated cell-cell adhesion opening and assembly, and activate the mitogen-activated protein kinase pathway (26). aPKC is associated with the tumorigenesis and progression of cancer. Murray *et al* (27) reported the progression of an aPKC λ/ι-derived colon adenoma to carcinoma, and that aPKC λ/ι is also necessary for APC/β-catenin mediated colon tumorigenesis. aPKC λ/ι expression in breast cancer is weak in ductal carcinoma *in situ*, and exhibits stronger staining in invasive ductal carcinoma (7). Furthermore, aPKC λ/ι overexpression in gastric cancer is a strong prognostic factor for cancer recurrence (3). The current study found that aPKC λ/ι expression becomes gradually stronger with progression from adenoma to invasive cancer. Although, the cancer cells that showed normal polarity, for example LST-CAs, showed weak expression for aPKC λ/ι, as observed in adenomas. However, the aPKC λ/ι expression became stronger in adenoma and early-stage cancer as a result of β-catenin accumulation in the nucleus and loss of BM. Furthermore, aPKC λ/ι expression was detected not only in the cytoplasm, but also in the nucleus. Perander *et al* also reported that while wild-type aPKC l is predominantly localized in the cytoplasm, two different point mutations in the catalytic domain lead to nuclear accumulation of full-length aPKC l (28).

These results suggested that LSTs of adenoma and cancer *in situ* exhibit almost normal polarity, expression and distribution of the BM, as well as β-catenin and E-cadherin. A change in the polarity of colorectal tumors in expansive growth may be associated with the expression of aPKC λ/ι. Therefore, further investigation of the expression of aPKC λ/ι in colorectal cancer is required.

## Figures and Tables

**Table I:** Clinicopathological characteristics of flat- and polypoid-type tumors.

A, Clinicopathological characteristics of LSTs and FD-CAs				

Characteristics	LST-A	LST-CA	FD-CA	P-value
n	15	9	13	
Age, years (mean ± SE)	63.2±4.2	62.1±6.2	63.9±6.8	NS
Gender, n (%)				
Male	9 (60.0)	6 (66.7)	7 (53.8)	NS
Female	6 (40.0)	3 (33.3)	6 (46.2)	
Diameter, mm (mean ± SE)	17.1±6.2	22.8±5.3	38.3±15.5	<0.005
LST subtype, n (%)				
G-type	11 (73.3)	4 (44.4)	-	NS
F-type	4 (26.7)	5 (63.6)	-	
Site (relative to splenic flexure), n (%)				
Proximal colon	5 (33.3)	5 (55.6)	8 (61.5)	NS
Distal colon	8 (53.3)	3 (33.3)	3 (23.1)	
Rectum	2 (13.4)	1 (11.1)	2 (15.4)	

B, Clinicopathological characteristics of polypoid-type tumors				

Characteristics	P-A	P-CA		P-value

n	11	9		
Age, years (mean ± SE)	64.7±8.5	57.7±7.7		NS
Gender, n (%)				
Male	9 (81.2)	7 (77.8)		NS
Female	2 (18.2)	2 (22.2)		
Diameter, mm (mean ± SE)	12.1±2.3	15.3±4.3		NS
Site (relative to splenic flexure), n (%)				
Proximal colon	4 (36.4)	2 (22.2)		NS
Distal colon	3 (27.3)	3 (33.3)		
Rectum	4 (36.4)	4 (44.4)		

LST, laterally spreading tumor; LST-A, LST, adenoma; LST-CA, LST, cancer in adenoma; FD-CA, flat depressed-type cancer; P-A, polypoid-type adenoma; P-CA, polypoid-type cancer in adenoma; SE, standard error; NS, not significant.

**Table II:** Expression of aPKC λ/ι, β-catenin, E-cadherin and type IV collagen in LSTs and polypoid-type tumors of each histological type.

A, aPKC λ/ι					

Type	LST-A, n (%)	LST-CA, n (%)	FD-CA, n (%)	P-A, n (%)	P-CA, n (%)
1+	13 (86.6)	5 (55.6)	0 (0.0)	5 (45.5)	0 (0.0)
2+	1 (6.7)	3 (33.3)	3 (23.1)	5 (45.5)	3 (33.3)
3+	1 (6.7)	1 (11.1)	10 (76.9)	1 (9.0)	6 (66.7)
Total	15 (100.0)	9 (100.0)	13 (100.0)	11 (100.0)	9 (100.0)

B, β-catenin					

Type	LST-A, n (%)	LST-CA, n (%)	FD-CA, n (%)	P-A, n (%)	P-CA, n (%)

P	13 (86.6)	7 (77.8)	1 (7.7)	10 (99)	2 (22.2)
C	2 (13.3)	1 (11.1)	4 (30.8)	1 (9)	2 (22.2)
N	0 (0)	1 (11.1)	8 (61.5)	0 (0)	5 (55.6)
Total	15 (100.0)	9 (100.0)	13 (100.0)	11 (100.0)	9 (100.0)

C, E-cadherin					

Type	LST-A, n (%)	LST-CA, n (%)	FD-CA, n (%)	P-A, n (%)	P-CA, n (%)

P	4 (26.7)	1 (11.1)	0 (0.0)	2 (18.2)	1 (11.1)
H	11 (73.3)	7 (77.8)	4 (30.8)	9 (81.8)	3 (33.3)
L	0 (0.0)	1 (11.1)	9 (69.2)	0 (0.0)	5 (55.6)
Total	15 (100.0)	9 (100.0)	13 (100.0)	11 (100.0)	9 (100.0)

D, Type IV collagen					

Type	LST-A, n (%)	LST-CA, n (%)	FD-CA, n (%)	P-A, n (%)	P-CA, n (%)

Cont	14 (93.3)	6 (66.7)	0 (0.0)	3 (27.3)	0 (0.0)
D	1 (6.7)	2 (22.2)	1 (7.6)	8 (72.7)	2 (22.2)
L	0 (0.0)	1 (11.1)	12 (92.4)	0 (0.0)	7 (77.8)
Total	15 (100.0)	9 (100.0)	13 (100.0)	11 (100.0)	9 (100.0)

aPKC, atypical protein kinase C; LST, laterally spreading tumor; LST-A, LST, adenoma; LST-CA, LST, cancer in adenoma; FD-CA, flat depressed-type cancer; P-A, polypoid-type adenoma; P-CA, polypoid-type cancer in adenoma; P, preserved type; C, cytoplasmic type; N, nucleic type; H, heterogeneous type; L, lost type; Cont, continuous type; D, discontinuous type.
